# A Systematic Study of Anti-Osteosarcoma Mechanism of pH-Sensitive Charge-Conversion Cinnamaldehyde Polymeric Prodrug Micelles In Vitro

**DOI:** 10.3390/biomedicines11061524

**Published:** 2023-05-25

**Authors:** Jiapeng Deng, Qichang Wang, Huihui Xu, Guoqing Li, Su Liu, Yixiao Chen, Fei Yu, Weiqiang Yan, Hui Zeng, Peng Liu

**Affiliations:** 1 National & Local Joint Engineering Research Center of Orthopaedic Biomaterials, Peking University Shenzhen Hospital, Shenzhen 518036, China; 2 Department of Bone & Joint Surgery, Peking University Shenzhen Hospital, Shenzhen 518036, China; 3 Department of Radiology, Peking University Shenzhen Hospital, Shenzhen 518036, China

**Keywords:** cinnamaldehyde prodrug, osteosarcoma targeting, PI3K/Akt, micelles

## Abstract

Osteosarcoma is an aggressive malignant neoplasm, and it is of great significance to the fabrication and investigation of the anti-tumor mechanism of nanomedicine in the treatment of osteosarcoma. Herein, a cinnamaldehyde polymeric prodrug micelle with pH-sensitive charge-conversion ability (mPEG-*b*-P(C7-*co*-CA)) was fabricated, and the anti-osteosarcoma mechanism of mPEG-*b*-P(C7-*co*-CA) micelle was investigated. mPEG-*b*-P(C7-*co*-CA) micelles were prepared by self-assembly method, and their diameter was 227 nm. mPEG-*b*-P(C7-*co*-CA) micelles could regulate the cell cycle and inhibit the proliferation of 143B cells, which was demonstrated by flow cytometry analysis, CCK-8 assay and 5-Ethynyl-2′-deoxyuridine (EdU) staining. The wound-healing assay and transwell assay showed that mPEG-*b*-P(C7-*co*-CA) micelles effectively inhibited the migration and invasion of 143B cells. It was proven that mPEG-*b*-P(C7-*co*-CA) micelles downregulated the levels of proliferation and apoptosis-related proteins and affected osteosarcoma migration and invasion by inhibiting the epithelial-mesenchymal transition (EMT). In addition, mPEG-*b*-P(C7-*co*-CA) micelles can also inhibit the transcriptional activity of the PI3K/Akt signaling pathway. Therefore, these findings provide new evidence for the pharmacological effects of mPEG-*b*-P(C7-*co*-CA) micelles.

## 1. Introduction

Traditional Chinese medicine is an important source for the discovery and development of cancer prevention and treatment drugs [1]. Cinnamaldehyde (CA) is a hydrophobic small molecule and is the main component of cinnamon bark essential oil (60–75%) [2]. It has a number of pharmacological effects, such as anti-oxidative stress, hypolipemia, anti-tumor and other effects [3,4]. CA has been found to achieve antitumor properties by selectively interfering with mitochondrial function and ROS levels in tumor cells [5]. However, the clinical application of CA is still limited due to its low aqueous solubility, instability, and poor bioavailability [6]. In addition, cinnamaldehyde has a short half-life in human plasma and lacks tumor-targeting properties, which also limits its pharmacological usage and bioavailability [7,8].

With the development of nanotechnology, a variety of nanomaterials, including liposomes, micelles, inorganic nanoparticles, etc. [9,10], have been designed and fabricated to achieve drug delivery for tumor treatment. Therefore, to extend the application of cinnamaldehyde, many cinnamaldehyde-loaded nano-based delivery systems have been developed. Liu et al. developed a tumor-targeted hyaluronic-acid-based oxidative stress nanoamplifier with ROS generation and GSH depletion through the targeted delivery of cinnamaldehyde (CA) and β-phenylethyl isothiocyanate (PEITC). This system can increase the ROS level in tumor cells and effectively kill tumor cells [11]. Wang et al. designed a pH-responsive small molecular nano-prodrug emulsified from the twin drugs of tryptamine and cinnamaldehyde for the targeted synergistic treatment of glioma [12]. Wani et al. reported a biocompatible functionalized magnetite-nanoparticles system for hyperthermia and the targeted delivery of cinnamaldehyde (CA) in breast cancer [13].

Osteosarcoma is a highly aggressive malignant bone tumor that usually occurs among teenagers and young adults [14]. Chemotherapy is one of the main treatments for osteosarcoma. Studies have shown that cinnamaldehyde can inhibit tumor spread and tumor cell invasion, affect the cell cycle and induce apoptosis, which made it become one of the candidates for osteosarcoma chemotherapy drugs [15]. The PI3K/Akt signaling pathway is considered to be one of the most important carcinogenic pathways in human cancer. Accumulating evidence indicates that this pathway is frequently overactivated in osteosarcoma and contributes to the occurrence and progression of tumors, including tumor growth, invasion, cell-cycle progression, inhibition of apoptosis, metastasis, and drug resistance [16].

It has been proven that the surface characteristics (size and surface charge) of nanoparticles play a key role in the interaction between nanoparticles and cells [17]. Tumor cells usually have a negative charge on their surface, and positively charged nanoparticles have a stronger affinity for tumor cells, thereby enhancing cell uptake [18]. However, positively charged nanoparticles are easily captured and cleared by the reticuloendothelial system because there are various negatively charged plasma proteins in the blood circulation [19]. Moreover, positively charged nanoparticles often produce nonspecific cytotoxicity to normal cells, resulting in serious side effects [20]. Therefore, positively charged nanoparticles are not the best candidates for drug-delivery carriers in vivo. In contrast, negatively charged nanoparticles can avoid being trapped by the reticuloendothelial system, thereby prolonging the blood circulation time and improving accumulation at tumor sites. In addition, due to the increase in lactic-acid production caused by the Warburg effect, the pH value of the tumor extracellular microenvironment (TEM) is approximately 6.5, which is far lower than the pH value (about 7.4) of the physiological environment (blood and normal tissue) [21]. In summary, the pH difference between tumors and the physiological environment can be used as a trigger for the charge reversal of nanoparticles, achieving a negative charge retention in the blood circulation (pH 7.4) and positive charge retention in tumor cells (pH 6.5).

**Scheme 1:**
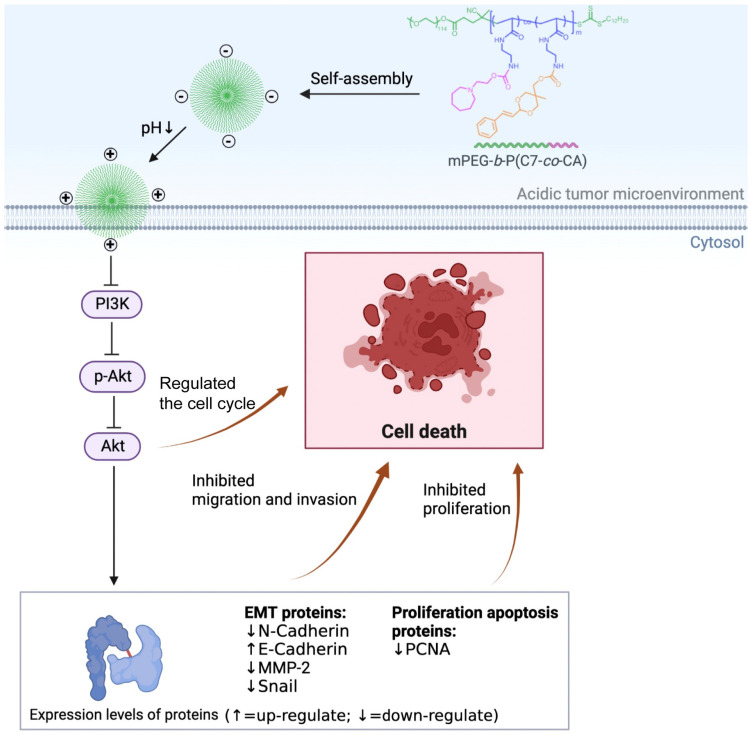
Schematic illustration of cinnamaldehyde polymeric prodrug micelles (mPEG-*b*-P(C7-*co*-CA)) inhibiting the proliferation, migration, and invasion of osteosarcoma 143B cells in vitro and leading to cell death.

A cinnamaldehyde polymeric prodrug micelle with pH-sensitive charge-conversion ability (mPEG-*b*-P(C7-*co*-CA)) was fabricated previously by our group for the effective chemotherapy of osteosarcoma through the targeted delivery of cinnamaldehyde [22]. Due to their pH-sensitive charge-conversion capability, mPEG-*b*-P(C7-*co*-CA) micelles have been demonstrated to be effective in targeting anti-osteosarcoma. However, the anti-osteosarcoma mechanism of mPEG-*b*-P(C7-*co*-CA) prodrug micelles is still unclear, and it is useful to promote its application by deeply understanding their anti-osteosarcoma mechanism. Therefore, a series of experiments were carried out to investigate its anti-osteosarcoma mechanism. This study indicated that mPEG-*b*-P(C7-*co*-CA) micelles can inhibit the growth, migration, and invasion of osteosarcoma cells in vitro, and the transcriptional activity of the PI3K/Akt signaling pathway of osteosarcoma cells was also inhibited by mPEG-*b*-P(C7-*co*-CA) micelles treatments (Scheme 1).

## 2. Materials and Methods

### 2.1. Cells and Materials

Human osteosarcoma cell lines 143B, MG63 and U2OS were purchased from the American Type Culture Collection (ATCC, Manassas, VA, USA). Cell Counting Kit-8 (CCK8) was purchased from Beyotime Biotechnology (Shanghai, China), and E-Click EdU Cell Proliferation Imaging Assay Kit was purchased from Elabscience Biotechnology Co., Wuhan, China. Transwell was purchased from Corning Corporation, Corning, NY, USA. Matrigel was purchased from BD Biosciences, San Jose, CA, USA. Cell Cycle Assay Kit was purchased from Dojindo Molecular Technologies, Inc. (Kumamoto, Japan). Horseradish peroxidase-labeled goat anti-rabbit and goat anti-mouse IgG (secondary antibody), mouse anti-human β-actin Cloned antibodies, PCNA, Bcl-2 monoclonal antibodies and rabbit anti-human N-cadherin, E-cadherin, MMP-2, Snail, PI3K, AKT, Phosphorylated AKT monoclonal antibodies were purchased from Cell Signaling Technology (CST, Danvers, MA, USA). mPEG-*b*-P(C7-*co*-CA) and mPEG-*b*-PC7A were prepared according to our previous report [22].

### 2.2. Preparation of mPEG-b-P(C7-co-CA) and mPEG-b-PC7A Micelles

Reconstitute mPEG-*b*-P(C7-*co*-CA) with DMF to 5 mg/mL, then drop 1 mL of mPEG-*b*-P(C7-*co*-CA) into 3 mL of dd H_2_O within 1 min while stirring. Stir at room temperature for 30 min, then transfer to dialysis tubes (MW cutoff: 3500) and dialyze for 24 h at room temperature. Afterwards, mPEG-*b*-P(C7-*co*-CA) micelles were stored at 4 °C. mPEG-*b*-PC7A micelles were prepared according to the same method.

### 2.3. Morphology, Particle Size and Zeta Potential of mPEG-b-P(C7-co-CA) Micelles

The morphology of mPEG-*b*-P(C7-*co*-CA) micelles was observed by transmission electron microscopy (TEM, TF20, FEI, Eindhoven, The Netherlands). The particle size of mPEG-*b*-P(C7-*co*-CA) micelles was measured by dynamic light scattering (DLS) on a Zetasizer (Nano-ZS90, Malvern Panalystic, Malverin, UK). In addition, the zeta potential of mPEG-*b*-P(C7-*co*-CA) micelles at pH 6.5 and 7.4 was also measured on the Zetasizer. Each sample was measured three times.

### 2.4. Cellular Uptake Assay

143B cells were seeded in 96-well plates at a density of 1 × 10^4^ cells per well. After the cells adhered to the wall, the medium was replaced with serum-free DMEM medium containing mPEG-*b*-P(C7-*co*-CA) micelles, which was loaded with coumarin 6 before its pH value was adjusted to 6.5 and 7.4, and the incubator continued to cultivate for 3 h. Subsequently, cells were fixed with 4% paraformaldehyde (Beyotime Biotechnology) for 20 min at RT. Finally, the nuclei were stained with DAPI for 15 min. Fluorescence images were observed with a fluorescence microscope (DMI8 LEICA), and a semi-quantitative analysis of fluorescence intensity was performed with Image J 1.5 (NIH, Bethesda, MD, USA).

### 2.5. Cell Cytotoxicity Assay

The 3-(4,5-dimethylthiazole-2-yl)-2,5-diphenyltetrazoli-umbromide (MTT) assay was used to investigate the cytotoxic effect of mPEG-*b*-P(C7-*co*-CA) micelles. 143B cells were seeded in 96-well plates at a density of 5 × 10^3^ cells per well. After the cells adhered to the wall, the medium was replaced with fresh DMEM medium at pH 6.5 or 7.4, which contained mPEG-*b*-P(C7-*co*-CA) micelles with different concentrations, and the culture was continued for 24 h. Afterwards, the medium was replaced with MTT (0.5 mg/mL) in fresh DMEM, and the incubator continued to cultivate for 4 h. The resulting crystal formazan was then solubilized in 100 μL 10% sodium dodecyl sulfate containing 0.01 M hydrochloric acid. Absorbance was measured at 595 nm using a microplate reader (Multiskan GO, Thermo Fisher Scientific, Shanghai, China). Use GraphPad Prism 8.0 to analyze IC50 values between different groups.

### 2.6. Cell Counting Kit-8 (CCK-8) Assay

143B, MG63 and U2OS cells were seeded in a 96-well plate at a density of 3 × 10^3^ cells per well. After the cells adhered to the wall, the medium was replaced with fresh DMEM medium at pH 6.5, which contained mPEG-*b*-PC7A or mPEG-*b*-P(C7-*co*-CA) micelles (12 µg/mL of cinnamaldehyde). After 0, 6, 12, 24, 48 and 72 h of cultivation, 10 μL CCK-8 reagent was added to each well followed by culture for 2 h at 37 °C in 5% CO_2_. Absorbance at 450 nm was measured using a microplate reader (Multiskan GO).

### 2.7. EdU Staining Assay

143B cells were seeded in 24-well plates at a density of 1 × 10^4^ cells per well. After the cells adhered to the wall, the medium was replaced with fresh DMEM medium at pH 6.5, which contained mPEG-*b*-PC7A or mPEG-*b*-P(C7-*co*-CA) micelles (12 µg/mL of cinnamaldehyde). After 24 h of incubation, cells were fixed with 4% paraformaldehyde (Beyotime Biotechnology) for 15 min at RT. Cell proliferation was monitored by EdU staining reagent (Elabscience Biotechnology) following the manufacturer’s instructions. Cells were observed by fluorescence microscopy (DMI8 LEICA), and then the positive rate (EdU/DAPI) of three random fields was calculated.

### 2.8. Cell-Cycle Analysis by Flow Cytometry

143B cells were seeded in 6-well plates at a density of 1 × 10^6^ cells per well. After the cells adhered to the wall, the medium was replaced with fresh DMEM medium at pH 6.5, which contained mPEG-*b*-PC7A or mPEG-*b*-P(C7-*co*-CA) micelles (12 µg/mL of cinnamaldehyde). After 24 h of incubation, the cell cycle was monitored by Cell Cycle Assay Kit (Dojindo China Co., Ltd., Shanghai, China) following the manufacturer’s instructions. Then, a flow cytometer (Becton Dickinson, Mountain View, CA, USA) was used to measure the cell cycle. The result was presented as a percentage.

### 2.9. Wound-Healing Assay

143B cells were seeded in 6-well plates and cultured to 90% confluence. Then, a pipette tip was used to scratch the cell culture plate vertically along the 6-well plate’s diameter. Then, the medium was discarded, and fresh medium (2 mL, pH 6.5) containing mPEG-*b*-PC7A or mPEG-*b*-P(C7-*co*-CA) micelles (12 µg/mL of cinnamaldehyde) was added. Images of the wound were captured after incubation for 12 and 24 h. The area change of the scratch area was calculated by Image J software.

### 2.10. Transwell Assays

The Matrigel solution and the medium were diluted at a ratio of 1:5 and evenly spread in the upper cavity of the transwell chamber, after which 3 × 10^4^ cells were added to the upper cavity of the transwell, and fresh medium containing mPEG-*b*-PC7A or mPEG-*b*-P(C7-*co*-CA) micelles (12 µg/mL of cinnamaldehyde) was added to the lower cavity of the transwell. After 24 h of incubation, it was stained with crystal violet and photographed. Matrigel was not added to the upper chamber to determine the cell-migration ability, and the experimental conditions were the same.

### 2.11. Western Blotting (WB)

143B cells were seeded in 6-well plates at a density of 1 × 10^6^ cells per well. After the cells adhered to the wall, the medium was replaced with fresh DMEM medium at pH 6.5, which contained mPEG-*b*-PC7A or mPEG-*b*-P(C7-*co*-CA) micelles (12 µg/mL of cinnamaldehyde). After 24 h, the cells were lysed, protein was extracted, and the BCA kit (Beyotime Biotechnology) was used to detect the protein concentration. After separation of the proteins by gradient SDS-PAGE, the proteins were transferred to PVDF membranes. After blocking with 5% nonfat milk for 1 h, the primary antibody was incubated overnight at 4 °C, and the secondary antibody was incubated at 37 °C for 1 h. Signals were examined using an ECL detection system (Bio-Rad, Hercules, CA, USA).

### 2.12. Statistical Analysis

All data are expressed as the mean ± standard deviation. Statistical analysis was performed by Prism 8.0 (GraphPad Software Inc., San Diego, CA, USA). One-way analysis of variance was used for differences between multiple groups, and Tukey’s test was used for comparison between groups. Differences were statistically significant at * *p* < 0.05, ** *p* < 0.01 and *** *p* < 0.001.

## 3. Results and Discussion

### 3.1. Characterization of mPEG-b-P(C7-co-CA) Micelles

**Figure 1:**
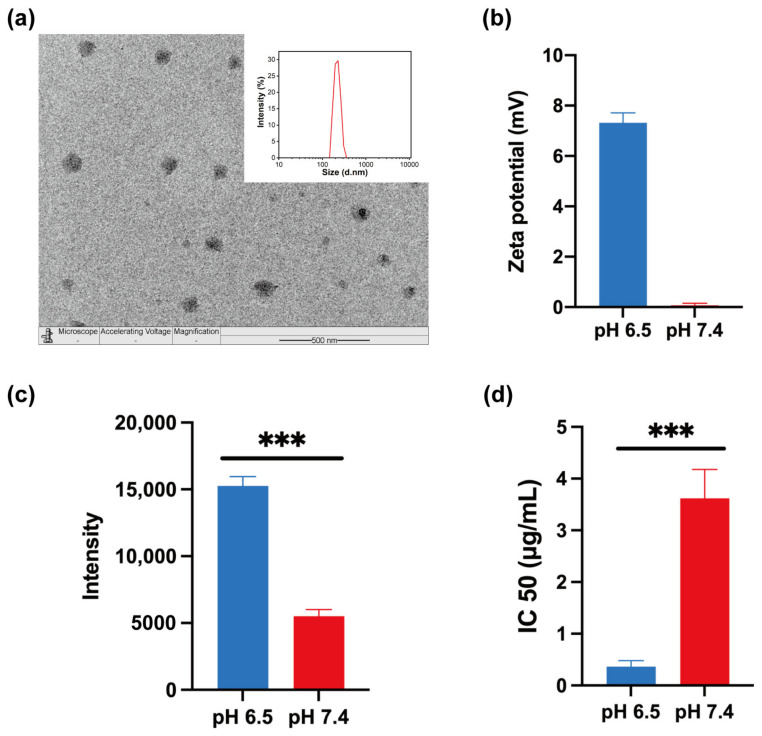
(**a**) Particle size distribution and TEM of mPEG-*b*-P(C7-*co*-CA) micelles; (**b**) zeta potential, (**c**) cellular uptake fluorescence semi-quantitative analysis, and (**d**) IC50 value of mPEG-*b*-P(C7-*co*-CA) micelles at different pH levels (scale bar 500 nm, *** *p* < 0.001, vs. the control group).

As amphiphilic block copolymer can self-assemble into micelles within aqueous solution, the mPEG-*b*-P(C7-*co*-CA) micelles with a spherical shape and a diameter of 227 nm were prepared by self-assembly of pH-sensitive amphiphilic cinnamaldehyde polymeric prodrug mPEG-*b*-P(C7-*co*-CA) (Figure 1a). This system presents an acid-induced charge-conversion property from a neutral to positive charge. The Zeta potential of mPEG-*b*-P(C7-*co*-CA) micelles is 0.13 mV at pH 7.4 (Figure 1b). However, the surface charge of mPEG-*b*-P(C7-*co*-CA) micelles increased significantly to 7.04 mV with the pH decreasing to 6.5 (Figure 1b). As mPEG-*b*-P(C7-*co*-CA) micelles can convert neutral charges on the surface to positive charges in an acidic tumor microenvironment (pH 6.5) while the surface charge of osteosarcoma cells is usually negative, mPEG-*b*-P(C7-*co*-CA) micelles can specifically target osteosarcoma 143B cells in vitro by electrostatic adsorption. Thus, mPEG-*b*-P(C7-*co*-CA) micelles showed a strong affinity to 143B cells and achieved an effective 143B cells target under an acidic environment (Figure 1c). Therefore, mPEG-*b*-P(C7-*co*-CA) micelles can achieve an effective anti-osteosarcoma with a lower IC50 value at pH 6.5 compared with that at pH 7.4 (Figure 1d).

### 3.2. Effects of mPEG-b-P(C7-co-CA) Micelles on the Proliferation of 143B Cells

To promote the clinical application of mPEG-*b*-P(C7-*co*-CA) micelles, it is necessary to investigate its anti-tumor mechanism in more detail. The uncontrolled proliferation of tumor cells is an important factor leading to poor prognosis of patients [23]. Herein, the effect of mPEG-*b*-P(C7-*co*-CA) micelles on the proliferation of 143B cells under acidic conditions (pH 6.5) was first investigated using CCK-8 and EdU methods. In order to exclude the influence of carrier material, we also prepared a blank micelles group, mPEG-*b*-PC7A micelles without CA, as the control of mPEG-*b*-P(C7-*co*-CA) micelles for investigation.

**Figure 2:**
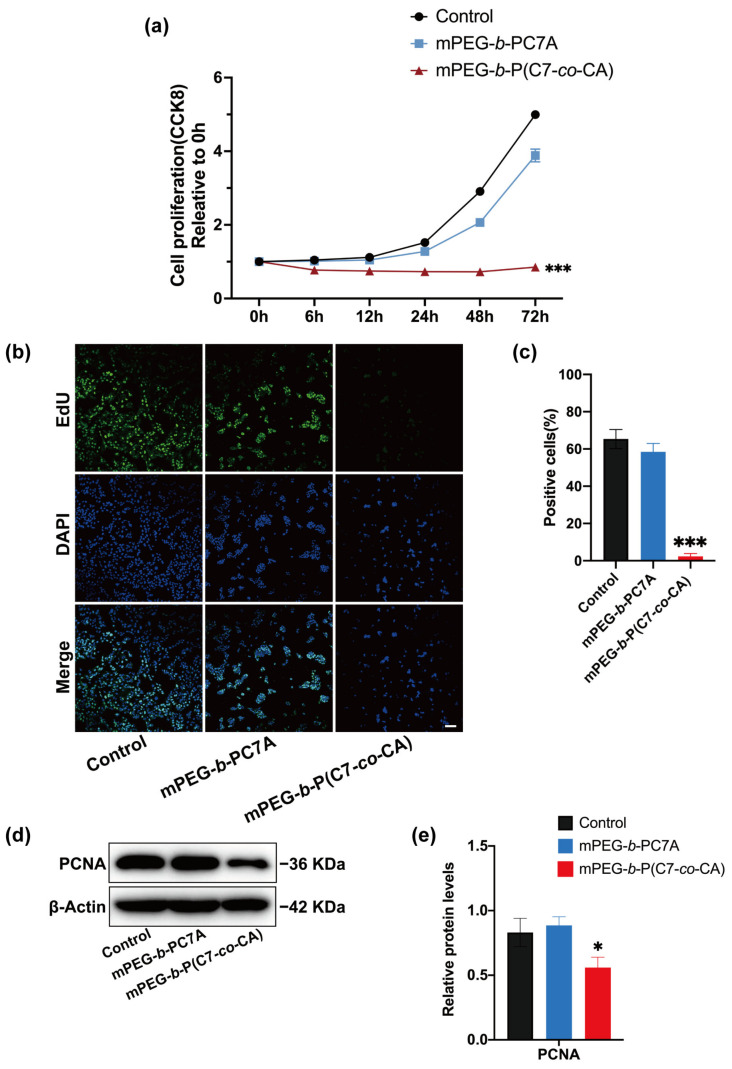
Effect of mPEG-*b*-P(C7-*co*-CA) micelles on the proliferation of 143B cells detected by (**a**) CCK-8 and (**b**,**c**) EdU assays. (**d**,**e**) The expression levels of proliferation-related protein PCNA detected by Western blotting (scale bar 100 µm, * *p* < 0.05, *** *p* < 0.001, vs. the control group).

As shown in Figure 2a, compared with the control and mPEG-*b*-PC7A micelles groups, mPEG-*b*-P(C7-*co*-CA) micelles significantly inhibited the proliferation of 143B cells in a time-dependent manner. In addition, we investigated the effect of mPEG-*b*-P(C7-*co*-CA) micelles on the proliferation of MG63 and U2OS cells, and the results showed that mPEG-*b*-P(C7-*co*-CA) micelles can also significantly inhibit the proliferation of MG63 and U2OS cells (Figure S1). Similar results were also observed by EdU method. EdU-labeled positive proliferating cells showed green fluorescence, and the proportion of positive cells in the control group and mPEG-*b*-PC7A group was higher than that in the mPEG-*b*-P(C7-*co*-CA) group (Figure 2b,c). In addition, we found that mPEG-*b*-P(C7-*co*-CA) micelles downregulated the protein levels of the proliferation-related protein, Proliferating Cell Nuclear Antigen (PCNA) (Figure 2d,e), which plays an important role in the initiation of cell proliferation [24]. Moreover, compared with the free CA, mPEG-*b*-P(C7-*co*-CA) micelles could inhibit the proliferation of 143B cells more effectively under the conditional treatment of the same CA concentration [25,26]. Compared with other nanodrugs, mPEG-*b*-P(C7-*co*-CA) micelles also exhibited superior anti-tumor effects [27]. Overall, these results indicate that mPEG-*b*-P(C7-*co*-CA) micelles have inhibitory effects on the proliferation of 143B cells in a weakly acidic environment. This is due to a better tumor endocytosis and more efficient delivery of CA in a weakly acidic environment.

### 3.3. Effects of mPEG-b-P(C7-co-CA) Micelles on 143B Cell Cycle

**Figure 3:**
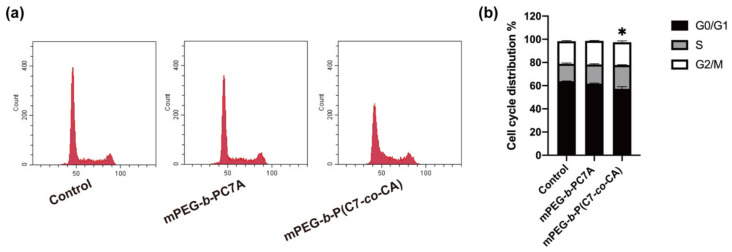
(**a**,**b**) Effect of mPEG-*b*-P(C7-*co*-CA) micelles on the cell cycle of 143B cells was detected by flow cytometry (* *p* < 0.05, vs. the control group).

Dysregulation of the cell cycle can lead to uncontrolled cell proliferation [28], and cell-cycle dysregulation can also induce apoptosis and make cells be part of tumor development and therapeutic responses [29]. To confirm the relationship between the growth inhibition and cell cycle, we performed a flow cytometry analysis. The results showed that mPEG-*b*-P(C7-*co*-CA) micelles caused a cell-cycle transition from the G0/G1 to S phase compared with the control and mPEG-*b*-PC7A groups (Figure 3a,b). These results indicate that the mPEG-*b*-P(C7-*co*-CA) micelles treatment effectively regulated the cell cycle in 143B cells.

### 3.4. Effects of mPEG-b-P(C7-co-CA) Micelles on Migration and Invasion of 143B Cells

**Figure 4:**
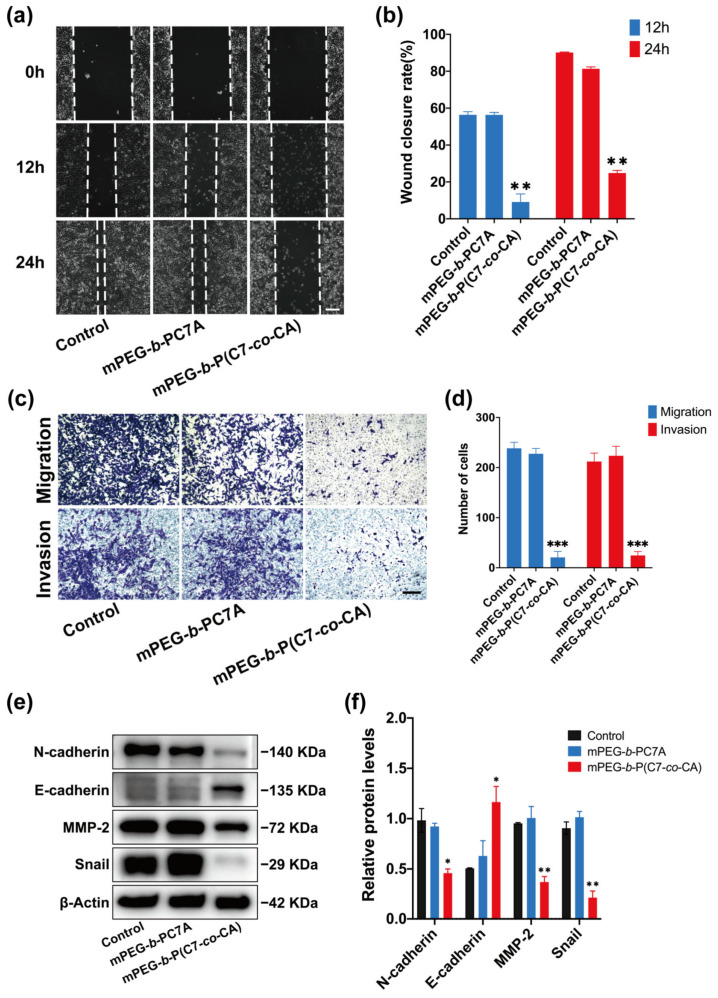
Effect of mPEG-*b*-P(C7-*co*-CA) micelles on migration and invasion of 143B cells was detected by (**a**,**b**) wound-healing assays and (**c**,**d**) transwell assay. (**e**,**f**) The expression levels of migration- and invasion-related proteins N-cadherin, E-cadherin, MMP-2 and Snail detected by Western blotting (scale bar 100 µm, * *p* < 0.05, ** *p* < 0.01, *** *p* < 0.001, vs. the control group).

Metastatic progression is the main cause of death in cancer patients, and osteosarcoma is prone to distant metastases [30]. Thus, it is important to study the anti-metastatic properties of mPEG-*b*-P(C7-*co*-CA) micelles. To investigate the effect of mPEG-*b*-P(C7-*co*-CA) micelles on the migration and invasion of 143B cells, transwell assays and wound-healing assays were performed. As shown in Figure 4a,b, compared with control and mPEG-*b*-PC7A micelles groups, mPEG-*b*-P(C7-*co*-CA) micelles significantly inhibited the cell migratory ability with the lowest wound-healing rate. Similar results were obtained in the transwell assay. These results demonstrated that mPEG-*b*-P(C7-*co*-CA) micelles not only inhibited the migration of 143B cells, but also inhibited the invasion of 143B cells (Figure 4c,d).

Western blot was also utilized to investigate the invasion- and migration-related proteins expression in 143B cells treated with mPEG-*b*-P(C7-*co*-CA) micelles. Epithelial-mesenchymal transition (EMT) is essential in tumor invasion and migration, and enables invasion to initiate tumor metastasis [31]. The abnormal expression of N-cadherin, E-cadherin and Snail is an important mechanism of EMT, which accelerates the invasion and metastasis of tumor cells [32]. Studies have shown that the zinc finger transcription factor Snail binds to the E-box of the E-cadherin promoter region, downregulates the expression of E-cadherin, and increases the expression of N-cadherin to induce EMT [33]. In addition, MMP-2 is a member of the matrix metalloproteinase family and plays a key role in the process of tumor metastasis. Furthermore, the level of MMP-2 increases with tumor progression. Thus, it is recognized as a key protein of invasion [34]. As shown in Figure 4e,f, mPEG-*b*-P(C7-*co*-CA) micelles decreased the expression of MMP-2 and EMT-related proteins N-cadherin and Snail and upregulated the expression of E-cadherin. These results suggest that mPEG-*b*-P(C7-*co*-CA) micelles affect the migration and invasion of osteosarcoma by inhibiting EMT signaling.

### 3.5. mPEG-b-P(C7-co-CA) Micelles Downregulated PI3K/Akt Signaling Pathway in 143B Cells

**Figure 5:**
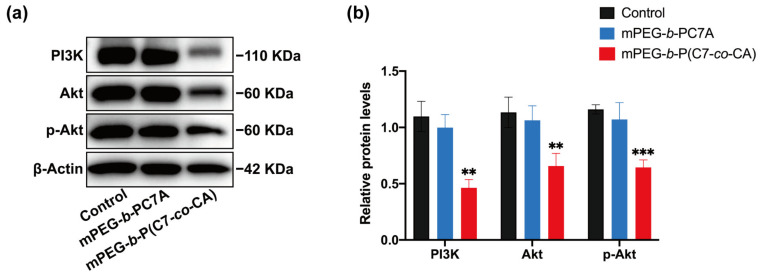
(**a**,**b**) The expression levels of PI3K/Akt signaling pathway-related proteins PI3K, Akt and p-Akt in 143B cells treated with mPEG-*b*-P(C7-*co*-CA) micelles were detected by western blotting (** *p* < 0.01, *** *p* < 0.001, vs. the control group).

The PI3K/Akt signaling pathway is a key pathway for regulating tumorigenesis, development, proliferation, and metastasis [35]. As a key regulator of tumor development, the PI3K/Akt pathway has become a potential target for traditional Chinese medicine therapy [36]. The activation of proteins associated with this pathway promotes the progression of osteosarcoma [35]. Therefore, we further studied the changes in the key proteins of the PI3K/Akt pathway after being treated with mPEG-*b*-P(C7-*co*-CA) micelles. The western blot analysis results suggested that the expression level of PI3K (a core member of the signaling pathway) was downregulated and that other related proteins, Akt and p-Akt, were also significantly downregulated after being treated with mPEG-*b*-P(C7-*co*-CA) micelles (Figure 5). The results proved that mPEG-*b*-P(C7-*co*-CA) micelles could inhibit the activation of the PI3K/Akt signaling pathway, thereby affecting the proliferation, migration, invasion, and cell cycle of osteosarcoma 143B cells.

## 4. Conclusions

In summary, mPEG-*b*-P(C7-*co*-CA) micelles can effectively inhibit the proliferation of 143B cells in an acidic tumor microenvironment. Moreover, mPEG-*b*-P(C7-*co*-CA) micelles can effectively regulate the cell cycle of 143B cells. In addition, prolonging the residence time of mPEG-*b*-P(C7-*co*-CA) micelles can inhibit the migration and invasion ability of 143B cells. Additionally, mPEG-*b*-P(C7-*co*-CA) micelles can downregulate the PI3K/Akt signaling pathway to achieve an anti-tumor effect. These experimental results showed that mPEG-*b*-P(C7-*co*-CA) micelles exhibit excellent anti-tumor effects in vitro.

## Data Availability

The data presented in this study are available on request from the corresponding author.

## Supplementary Materials

The following supporting information can be downloaded at: https://www.mdpi.com/article/10.3390/biomedicines11061524/s1, Figure S1: Effect of mPEG-b-P(C7-co-CA) micelles on the proliferation of MG63 cells (a) and U2OS 13 cells (b) detected by CCK-8 assay (*** *p* <0.001, vs the control group).

## References

[1] Ling C.Q., Yue X.Q., Ling C. (2014). Three advantages of using traditional Chinese medicine to prevent and treat tumor. J. Integr. Med..

[2] Sim J.X.F., Khazandi M., Pi H., Venter H., Trott D.J., Deo P. (2019). Antimicrobial effects of cinnamon essential oil and cinnamaldehyde combined with EDTA against canine otitis externa pathogens. J. Appl. Microbiol..

[3] Kostrzewa T., Przychodzen P., Gorska-Ponikowska M., Kuban-Jankowska A. (2019). Curcumin and Cinnamaldehyde as PTP1B Inhibitors with Antidiabetic and Anticancer Potential. Anticancer. Res..

[4] Gulec Peker E.G., Kaltalioglu K. (2021). Cinnamaldehyde and eugenol protect against LPS-stimulated oxidative stress and inflammation in Raw 264.7 cells. J. Food Biochem..

[5] Ka H., Park H.J., Jung H.J., Choi J.W., Cho K.S., Ha J., Lee K.T. (2003). Cinnamaldehyde induces apoptosis by ROS-mediated mitochondrial permeability transition in human promyelocytic leukemia HL-60 cells. Cancer Lett..

[6] Liu Z., Robinson J.T., Sun X., Dai H. (2008). PEGylated nanographene oxide for delivery of water-insoluble cancer drugs. J. Am. Chem. Soc..

[7] Lee K., Kwon B.M., Kim K., Ryu J., Oh S.J., Lee K.S., Kwon M.G., Park S.K., Kang J.S., Lee C.W. (2009). Plasma pharmacokinetics and metabolism of the antitumour drug candidate 2′-benzoyloxycinnamaldehyde in rats. Xenobiotica.

[8] Olsen R.V., Andersen H.H., Møller H.G., Eskelund P.W., Arendt-Nielsen L. (2014). Somatosensory and vasomotor manifestations of individual and combined stimulation of TRPM8 and TRPA1 using topical L-menthol and trans-cinnamaldehyde in healthy volunteers. Eur. J. Pain..

[9] Ferrari M. (2005). Cancer nanotechnology: Opportunities and challenges. Nat. Rev. Cancer.

[10] Pérez-Herrero E., Fernández-Medarde A. (2015). Advanced targeted therapies in cancer: Drug nanocarriers, the future of chemotherapy. Eur. J. Pharm. Biopharm..

[11] Liu Q., Ding X., Xu X., Lai H., Zeng Z., Shan T., Zhang T., Chen M., Huang Y., Huang Z. (2022). Tumor-targeted hyaluronic acid-based oxidative stress nanoamplifier with ROS generation and GSH depletion for antitumor therapy. Int. J. Biol. Macromol..

[12] Wang Z., Yao J., Guan Z., Wu H., Cheng H., Yan G., Tang R. (2021). pH-triggered small molecule nano-prodrugs emulsified from tryptamine-cinnamaldehyde twin drug for targeted synergistic glioma therapy. Colloids Surf. B Biointerfaces.

[13] Wani K.D., Kadu B.S., Mansara P., Gupta P., Deore A.V., Chikate R.C., Poddar P., Dhole S.D., Kaul-Ghanekar R. (2014). Synthesis, characterization and in vitro study of biocompatible cinnamaldehyde functionalized magnetite nanoparticles (CPGF Nps) for hyperthermia and drug delivery applications in breast cancer. PLoS ONE.

[14] Lee J.A., Lim J., Jin H.Y., Park M., Park H.J., Park J.W., Kim J.H., Kang H.G., Won Y.-J. (2021). Osteosarcoma in adolescents and young adults. Cells.

[15] Chu S.-C., Hsieh Y.-S., Hsu L.-S., Lin C.-Y., Lai Y.-A., Chen P.-N. (2022). Cinnamaldehyde decreases the invasion and u-PA expression of osteosarcoma by down-regulating the FAK signalling pathway. Food Funct..

[16] Zhang J., Yu X.-H., Yan Y.-G., Wang C., Wang W.-J. (2015). PI3K/Akt signaling in osteosarcoma. Clin. Chim. Acta.

[17] Arvizo R.R., Miranda O.R., Thompson M.A., Pabelick C.M., Bhattacharya R., Robertson J.D., Rotello V.M., Prakash Y., Mukherjee P. (2010). Effect of nanoparticle surface charge at the plasma membrane and beyond. Nano Lett..

[18] Fang Z., Pan S., Gao P., Sheng H., Li L., Shi L., Zhang Y., Cai X. (2020). Stimuli-responsive charge-reversal nano drug delivery system: The promising targeted carriers for tumor therapy. Int. J. Pharm..

[19] Liu L., Hitchens T.K., Ye Q., Wu Y., Barbe B., Prior D.E., Li W.F., Yeh F.-C., Foley L.M., Bain D.J. (2013). Decreased reticuloendothelial system clearance and increased blood half-life and immune cell labeling for nano-and micron-sized superparamagnetic iron-oxide particles upon pre-treatment with Intralipid. Biochim. Biophys. Acta Gen. Subj..

[20] Bernkop-Schnürch A. (2018). Strategies to overcome the polycation dilemma in drug delivery. Adv. Drug Deliv. Rev..

[21] Wu W., Luo L., Wang Y., Wu Q., Dai H.-B., Li J.-S., Durkan C., Wang N., Wang G.-X. (2018). Endogenous pH-responsive nanoparticles with programmable size changes for targeted tumor therapy and imaging applications. Theranostics.

[22] Deng J., Liu S., Li G., Zheng Y., Zhang W., Lin J., Yu F., Weng J., Liu P., Hui Z. (2023). pH-sensitive charge-conversion cinnamaldehyde polymeric prodrug micelles for effective targeted chemotherapy of osteosarcoma in vitro. Front. Chem..

[23] Xu X., Zhang M., Xu F., Jiang S. (2020). Wnt signaling in breast cancer: Biological mechanisms, challenges and opportunities. Mol. Cancer.

[24] Jayaraman S., Pazhani J., PriyaVeeraraghavan V., Raj A.T., Somasundaram D.B., Patil S. (2022). PCNA and Ki67: Prognostic proliferation markers for oral cancer. Oral Oncol..

[25] Huang Y., Chen J., Yang S., Tan T., Wang N., Wang Y., Zhang L., Yang C., Huang H., Luo J. (2020). Cinnamaldehyde Inhibits the Function of Osteosarcoma by Suppressing the Wnt/β-Catenin and PI3K/Akt Signaling Pathways. Drug Des. Dev. Ther..

[26] Lin C.Y., Hsieh Y.S., Chu S.C., Hsu L.S., Huang S.C., Chen P.N. (2022). Reduction of invasion and cell stemness and induction of apoptotic cell death by Cinnamomum cassia extracts on human osteosarcoma cells. Environ. Toxicol..

[27] Wei H., Chen J., Wang S., Fu F., Zhu X., Wu C., Liu Z., Zhong G., Lin J. (2019). A Nanodrug Consisting Of Doxorubicin And Exosome Derived From Mesenchymal Stem Cells For Osteosarcoma Treatment In Vitro. Int. J. Nanomed..

[28] Howe L.R., Brown A.M. (2004). Wnt signaling and breast cancer. Cancer Biol. Ther..

[29] Brown J.A., Yonekubo Y., Hanson N., Sastre-Perona A., Basin A., Rytlewski J.A., Dolgalev I., Meehan S., Tsirigos A., Beronja S. (2017). TGF-β-Induced Quiescence Mediates Chemoresistance of Tumor-Propagating Cells in Squamous Cell Carcinoma. Cell Stem Cell.

[30] Yang C., Tian Y., Zhao F., Chen Z., Su P., Li Y., Qian A. (2020). Bone microenvironment and osteosarcoma metastasis. Int. J. Mol. Sci..

[31] Tsai J.H., Yang J. (2013). Epithelial-mesenchymal plasticity in carcinoma metastasis. Genes Dev..

[32] Abd ElMoneim H.M., Zaghloul N.M. (2011). Expression of E-cadherin, N-cadherin and snail and their correlation with clinicopathological variants: An immunohistochemical study of 132 invasive ductal breast carcinomas in Egypt. Clinics.

[33] Papiewska-Pająk I., Kowalska M.A., Boncela J. (2016). Expression and activity of SNAIL transcription factor during Epithelial to Mesenchymal Transition (EMT) in cancer progression. Adv. Hyg. Exp. Med..

[34] Xu Y., Zhang J., Liu X., Huo P., Zhang Y., Chen H., Tian Q., Zhang N. (2019). MMP-2-responsive gelatin nanoparticles for synergistic tumor therapy. Pharm. Dev. Technol..

[35] Xu Z., Han X., Ou D., Liu T., Li Z., Jiang G., Liu J., Zhang J. (2020). Targeting PI3K/AKT/mTOR-mediated autophagy for tumor therapy. Appl. Microbiol. Biotechnol..

[36] Liu F., Xu J., Yang R., Liu S., Hu S., Yan M., Han F. (2023). New light on treatment of cervical cancer: Chinese medicine monomers can be effective for cervical cancer by inhibiting the PI3K/Akt signaling pathway. Biomed. Pharmacother..

