# Supplementary material for: A Systematic Study of Anti-Osteosarcoma Mechanism of pH-Sensitive Charge-Conversion Cinnamaldehyde Polymeric Prodrug Micelles In Vitro
Source: Biomedicines. 2023 May 25;11(6):1524. doi: 10.3390/biomedicines11061524 (PMC10295571; doi:10.3390/biomedicines11061524)
Supplement: Supplementary file 1 [file biomedicines-11-01524-s001.zip › biomedicines-2322625-supplementary.pdf]

biomedicines

MDPI

Supplementary Material

A systematic study of anti-osteosarcoma mechanism of pH-sensitive charge-conversion cinnamaldehyde polymeric prodrug micelles *in vitro*

Jiapeng Deng,[†,1,2] Qichang Wang,[†,1,2] Huihui Xu,[1,2] Guoqing Li,[1,2] Su Liu,[1,2] Yixiao Chen,[1,2] Fei Yu,[1,2] Weiqiang Yan,[3] Hui Zeng,[*,1,2] Peng Liu[*,1,2]

[1] National & Local Joint Engineering Research Center of Orthopaedic Biomaterials, Peking University Shenzhen Hospital, Shenzhen, PR China, 518036.
[2] Department of Bone & Joint Surgery, Peking University Shenzhen Hospital, Shenzhen, PR China, 518036.
[3] Department of Radiology, Peking University Shenzhen Hospital, Shenzhen, PR China, 518036.
† These authors contributed equally to this work.
* Correspondence: liupeng_polymer@126.com (P.L.); zenghui_36@163.com (H.Z.)

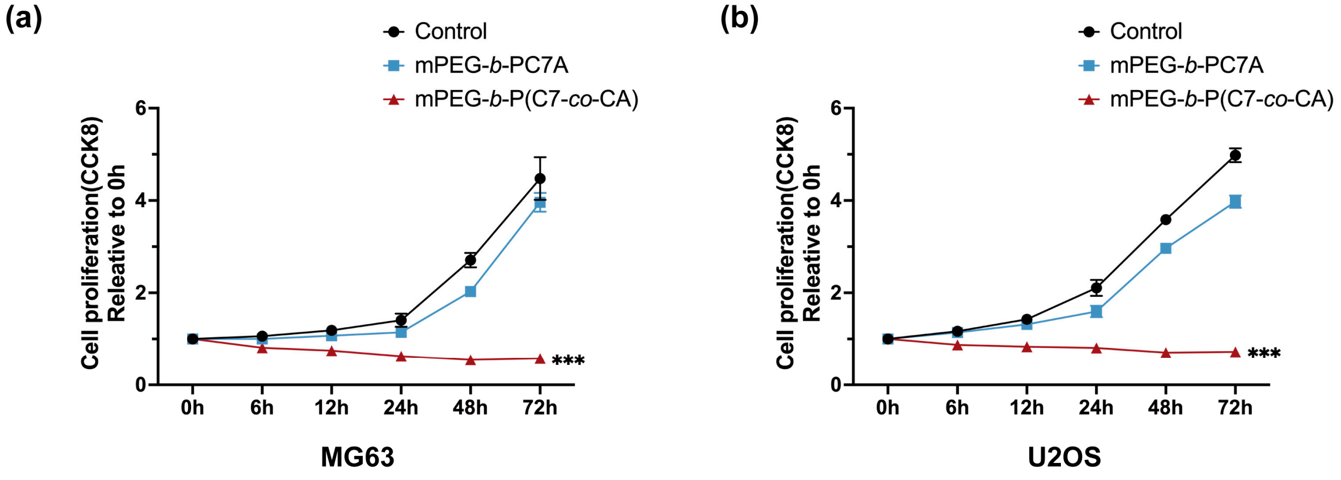

Figure S1. Effect of mPEG-*b*-P(C7-*co*-CA) micelles on the proliferation of MG63 cells (a) and U2OS cells (b) detected by CCK-8 assay (****P*<0.001, vs the control group).
